# The association between perception of health during pregnancy and the risk of cardiovascular disease: a prospective study

**DOI:** 10.1186/s40064-015-1639-6

**Published:** 2016-01-04

**Authors:** Hanis Hanum Zulkifly, Alexandra Clavarino, Yaman Walid Kassab, Kaeleen Dingle

**Affiliations:** Department of Pharmacy Practice, Faculty of Pharmacy, Universiti Teknologi MARA (UiTM), Kampus Puncak Alam, Puancak Alam Campus, 42300 Bandar Puncak Alam, Selangor Malaysia; Pharmacy Australia Centre of Excellence, University of Queensland, Woolloongabba, Brisbane, QLD 4102 Australia; Department of Hospital and Clinical Pharmacy, No. 3410, Jalan Teknokrat 3, Cyber 4, 63000 Cyberjaya, Selangor Darul Ehsan Malaysia; School of Public Health and Social Work, Faculty of Health, Queensland University of Technology, Kelvin Grove Campus, Brisbane, QLD 4059 Australia

**Keywords:** Self-perception, Health, Pregnancy, Cardiovascular disease, Longitudinal study

## Abstract

There is some evidence that self-rated perceptions of health are predictive of objective health outcomes, including cardiovascular disease, and mortality. The objective of this study was to examine the prospective association between perceptions of health during pregnancy and cardiovascular risk factors of mothers 21 years after the pregnancy. Data used were from the Mater University Study of Pregnancy (MUSP), a community-based prospective birth cohort study begun in Brisbane, Australia, in 1981. Logistic regression analyses were conducted. Data were available for 3692 women. Women who perceived themselves as not having a straight forward pregnancy had twice the odds (adjusted OR 2.0, 95 % CI 1.1–3.8) of being diagnosed with heart disease 21 years after the pregnancy when compared with women with a straight forward pregnancy (event rate of 5.2 versus 2.6 %). Women who experienced complications (other than serious pregnancy complications) during their pregnancy were also at 30 % increased odds (adjusted OR 1.3, 95 % CI 1.0–1.6) of having hypertension 21 years later (event rate of 25.7 versus 20 %). As a whole, our study suggests that pregnant women who perceived that they had complications and did not have a straight forward pregnancy were likely to experience poorer cardiovascular outcomes 21 years after that pregnancy.

**Electronic supplementary material:**

The online version of this article (doi:10.1186/s40064-015-1639-6) contains supplementary material, which is available to authorized users.

## Background

There is some evidence to suggest that self-rated perceptions of health are predictive of objective health outcomes, including cardiovascular diseases, and mortality (Bosworth et al. 1999; Idler and Benyamini 1997; Miilunpalo et al. 1997). People’s self-rated perceptions of their health, appear to be based on highly selective and on different aspects of their lives, including their objective health both mental and physical, their functioning and experience of any symptoms (Walke et al. 2007). One important determinant of health perception is age; with perceptions of good health declining with age (Australian Institute of Health and Welfare (AIHW) 2012). Cardiovascular disease (CVD) is an umbrella term that is commonly used to describe pathological conditions of the heart and blood vessels (National Vascular Disease Prevention Alliance 2012. CVDs include conditions such as coronary heart disease, cerebrovascular disease, heart failure, rheumatic heart disease and hypertension (National Vascular Disease Prevention Alliance 2012). Patients with diabetes, kidney disease, or hypercholesterolemia are at increased risk of developing CVDs. A recent meta-analysis of five case-control and 10 cohort studies found that, relative to women with uncomplicated pregnancies, women with a history of pre-eclampsia/eclampsia, had an increased risk of subsequent cardiac disease (in both case control and cohort studies) approximately double that of women with uncomplicated pregnancies (McDonald et al. 2008). CVDs have remained the leading cause of death in Australia over the last 10 years and continued to be the leading cause in 2011, accounting for 39 % of female deaths (Australian Institute of Health and Welfare (AIHW) 2012).

To date no studies have looked into women’s perception of health during pregnancy and their risk of developing cardiovascular diseases later on. Thus, the aim of this study was to assess women’s self-reported perceptions of health during pregnancy and their risk of cardiovascular diseases 21 years after pregnancy.

## Methods

The Mater-University of Queensland Study of Pregnancy (MUSP) is a prospective pregnancy cohort study consisting of 6703 women, aged 15–35 (mean age 26), who gave birth to a singleton child at a major public hospital in Brisbane between 1981 and 1984. At enrolment, women were at an average gestational age of 18 weeks (SD = 6.1). The cohort was followed prospectively both mother and child were assessed at the first antenatal visit, 3–5 days post birth, then 6 months, 5, 14 and 21 years after birth. At each follow-up phase (FU), data relating to maternal socioeconomic status, lifestyle, mental and physical health and family structure were collected.

The main analyses were restricted to 3692 women who reported perceptions of their health during their pregnancy and had information on cardiovascular diseases at the 21 year follow-up. Women who developed serious complications during the pregnancy were excluded from these analyses. Complete details of all exclusions are provided below. Written informed consent was obtained from the participants at all data collection phases. Ethical clearance has been obtained from the Mater Hospital and the University of Queensland for each stage of this study.

### Outcome variables

The primary outcome of this study was medically diagnosed cardiovascular disease as reported by women 21 years after the birth of their child. At the 21 year follow-up, women were asked three separate questions: “have you ever been told by a doctor (including during pregnancy period) that you have heart disease—hypertension—stroke?” Responses to these items were coded as either ‘yes’ or ‘no’.

Detailed obstetric information on their pregnancy, including any medical complications associated with the pregnancy, was available. Women who developed serious complications during the pregnancy, including hypertension (n = 53), pre-eclampsia (n = 337) and other gestational disorders such as urinary tract infection, cervical incompetence, blood disorders, and migraine (n = 141), were excluded from further analysis. A high risk pregnancy was defined as either hypertensive disorder of pregnancy (HDP) or gestational diabetes and was ascertained by consultant obstetricians associated with the study. Definitions have changed over time, at the time of this study (that is the early 1980’s) HDP was defined as having a diastolic blood pressure over 90 mmHg on at least two occasions beyond 20 weeks gestation associated with proteinuria and or excessive fluid retention (defined here as generalized oedema, including the face and hands and excessive weight gain) (Callaway et al. 2011). Serious complications of pregnancy have been linked to an increased risk of cardiovascular disease. Studies have found a doubling of risk for essential hypertension, ischaemic heart disease and stroke over time for women with pre-eclampsia (Bellamy et al. 2007; Callaway et al. 2013). For this reason these women were excluded from further analyses. The number of women that reported ever being diagnosed with a stroke at the 21-year FU was low (n = 57). These numbers were insufficient to adequately power the final model given the number of covariates assessed; therefore the results are not reported here.

### Exposure variables

#### Perceptions of health during pregnancy

The main exposure examined in this study is related to women’s perceptions of their own health during their pregnancy. At 3–5 days after the birth women were asked three questions about their overall health during their pregnancy: firstly if they felt generally unwell, this item was dichotomised to either generally feeling well or generally feeling unwell, secondly if the ‘pregnancy was straight forward’ and thirdly, if they had ‘some complications during this pregnancy’. Respondents answered the last two questions using a five-point Likert scale: (strongly agree, agree, neutral, disagree, and strongly disagree). The responses related to whether or not the pregnancy was straight forward were recoded to yes (strongly agree, agree and neutral) and no (disagree and strongly disagree). The responses from the question if they experienced complications during the pregnancy were recoded to yes (strongly agree, agree) and no (neutral, disagree, and strongly disagree).

### Potential confounders

#### Demographics characteristics

The following demographic characteristics were assessed as potential confounders and adjusted for consecutively: age, parity and education level were measured at their pregnancy, and family income was measured concurrently at the 21-year FU. Maternal age at delivery and parity were obtained from the obstetric record; the level of maternal education (did not complete secondary school, completed secondary school, completed further/higher education) was recorded at the initial antenatal visit. Family income was measured at the 21 year FU and was described by quintiles; these are based on the distribution of the midpoint of each income category and were categorized as low income (less than $399 per week) and normal income ($400–$1500 per week) (Australian Bureau of Statistics 2004).

#### Martial relationship and quality factors

Information on marital status at initial interview and at 21 year FU was grouped as currently single (single, separated, divorced or widowed) and live-in relationship (living together or married). The quality of the marital or similar relationship was assessed at the 21-year FU using the Spanier Dyadic Adjustment Scale (DAS-short form) (Spanier 1976). The reliability and validity of the DAS has been well established (Davis et al. 1998). Women were categorised as being in a relationship characterised either by conflict, or not; therefore, women reporting no conflict had relationships marked by a low frequency of quarrels and negative interactions, and little or no discussion of separation.

### Potential mediators

#### Clinical mediators

A number of clinical mediators were assessed, including BMI and menopausal status. BMI was calculated from height and weight measured at 21 year FU and pre-pregnancy. BMI was self-reported at the first antenatal visit. The BMI categories were based on World Health Organization (WHO) guidelines (National Health and Medical Research Council (NHMRC) 2013). Information on menopausal status was obtained at the 21 year FU. An indicator menopausal status was created using four variables that assessed timing and nature of menstrual periods and if women ever had surgery to remove their uterus or both ovaries. Menopausal status was defined as follows: pre-menopausal (bleeding in the past 3 months and with the same or increasing regularity as in the past year), peri-menopausal (bleeding in the past 12 months but not 3 months or decreased regularity compared with the previous year), post menopausal (no reported menstrual bleeding in the past 12 months) and menopause due to surgery (hysterectomy with or without ovarian conservation and or removal of both ovaries) (Tom et al. 2010).

#### Mental health mediators

For mental health characteristics, information on the onset, and the number of episodes of depression was obtained at each phase of the study (early pregnancy, 3–5 days after the birth, 6 months, 5-, 14- and 21 years FU). Depression was assessed using the seven item depression subscale from the Delusions Symptoms-States Inventory: State of Anxiety and Depression (DSSI) (Bedford and Foulds 1977). This measure was developed to detect signs and symptoms of psychopathology that limit a person’s capacity to function and maintain relationships. This measure has been validated (Bedford 1978), correlates well and shares item with other commonly used measures of depression and anxiety, such as the Edinburgh Postnatal Depression Scale and the Hospital Anxiety and Depression Scale (HADS) (Najman et al. 2000). In this study, women who reported experiencing four or more out of possible seven symptoms were considered to represent a case of depression. To facilitate interpretation this was dichotomised into those women who were considered to be never or ever depressed. The measure of anxiety used the seven item Anxiety subscale of the DSSI and the anxiety scale was similarly constructed.

#### Lifestyle mediators

Alcohol use and cigarette smoking were considered to be lifestyle mediators. Women were asked at each phase if they were currently smokers and the number of cigarettes smoked in the previous week. A composite alcohol consumption variable was created with three categories (abstainer, light, and moderate to heavy drinker).

### Statistical analyses

All analyses were performed using SPSS statistical software version 20 (SPSS Inc., Chicago, IL, USA). *P* values <0.05 were indicated statistical significance. Descriptive statistics were used to describe demographic, social, lifestyle, physical and mental health characteristics of the patients and their perceptions of health during pregnancy. Associations between perceptions of health during pregnancy and later cardiovascular outcomes (heart disease, hypertension and stroke) were obtained using Chi square tests.

Chi square tests and logistic regression were used to explore the bivariate associations between perception of health during pregnancy and later cardiovascular outcomes. A series of step-wise multivariate logistic regression models were progressively fitted and then manually assessed using backward selection. Associations with each outcome were adjusted consecutively for early pregnancy factors (model 2), demographics factors measured at the 21-year phase (model 3), clinically relevant factors BMI and mental health (model 4) and known mediators (model 5). This was undertaken separately for each of the cardiovascular outcomes. Non-significant factors (*p* value >0.1) were progressively removed, then the final model was reassessed until only significant effects (*p* value <0.05) remained. Unadjusted and adjusted odds ratios (OR) and 95 % confidence intervals were used to estimate the associations.

An attrition analysis found that women lost to follow-up were more likely to have some of the following characteristics: be teenage mothers; have a lower educational status; single or cohabitating and have three or more children; have used tobacco and alcohol during their pregnancy; and been depressed and anxious at the first antenatal visit (Najman et al. 2005).

## Results

Table 1 shows the baseline characteristics for the 3692 women included in the study. The mean age for women at entry was 26 years (SD 5.0), and close to three-quarters of the women interviewed had parity of one, the current pregnancy (n = 1603, 43.4 %), or two (n = 1090, 29.5 %). At the 21 year FU women were an average age of 46 years (SD 5.0). Approximately 70 % of the sample were either pre or peri-menopausal. Majority of women had a normal BMI both pre pregnancy and 21 years later. In this study, only 26.9 % of women reported ever being diagnosed with hypertension, a major risk factor for future cardiovascular disease (Additional file 1: Table S1. Reports bivariate results). Women were asked about their health during the pregnancy, 8.4 % of cohort reported feeling unwell and 28.6 % had some minor complications during their pregnancy (see Table 2). Women reporting more serious complications i.e. essential hypertension and pre-eclampsia were excluded from this analysis and subsequent analyses.

**Table 1:** Characteristics of the women 21 years after pregnancy, n = 3692

Demographics	Number	Percentage %
Age at study entry (years)	25.9 (SD = 5.0)	
Age 21-years after birth (years)	46 (SD = 5.0; range 34–67)	
Parity	2 children (IQR = 2)	43.4
Maternal education (*missing* = 25)		
Incomplete high school	581	15.8
Complete high school	2354	64.2
Post high school	732	20
Family income at 21-year phase (*missing* = 127)		
Low income (<$399/week)	747	21
Middle to high income ($400–$1500/week)	2818	79

Social factors		
Marital status at FCV (*missing* = 25)		
Currently single	389	10.6
Live in relationship	3278	89.4
Marital status at 21-year phase (*missing* = 25)		
Currently single	921	25.1
Live in relationship	2743	74.9
Dyadic adjustment post natal to 5 years (*missing* = 1109)		
Good	1718	66.5
Some conflict	338	13.1
Conflict	235	9.1
No partner	292	11.3
Dyadic adjustment at 21-year phase (*missing* = 35)		
Good	2265	61.9
Some conflict	535	14.6
Conflict	124	3.4
No partner	733	20

Lifestyle factors		
Smoking at all phases (*missing* = 12)		
Never smoked	1620	44
Ever smoked	2060	56
Alcohol consumption at 21-year phase (*missing* = 21)		
Abstainer	510	13.9
Light drinker	2158	58.8
Moderate to heavy drinker	1003	27.3

Physical health		
Pre pregnancy BMI (kg/m^2^) (*missing* = 354)		
Underweight (<18.5)	448	13.4
Normal (18.5–24.9)	2352	70.5
Overweight (25.0–29.9)	387	11.6
Obese (30.0–34.9)	107	3.2
Severe obesity (>35.0)	44	1.3
BMI at 21-year phase (kg/m^2^) (*missing* = 1762)		
Underweight (<18.5)	25	1.3
Normal (18.5–24.9)	690	35.8
Overweight (25.0–29.9)	594	30.8
Obese (30.0–34.9)	361	18.7
Severe obesity (>35.0)	260	13.5
Menopausal status at 21-year phase (*missing* = 44)		
Premenopausal	1253	34.3
Perimenopausal	1299	35.6
Postmenopausal	543	14.9
Surgical	553	15.2

Mental health		
No of episodes of anxiety at all phases (*missing* = 693)		
No anxiety	1753	58.5
One episode	634	21.1
Two episodes	335	11.2
Three episodes	182	6.1
Four episodes	95	3.2
No of episodes of depression at all phases (*missing* = 1305)		
No depression	1744	73.1
One episode	509	21.3
Two episodes	110	4.6
Three episodes	23	1.0
Four episodes	1	0.0

**Table 2:** Perceptions of health during pregnancy and primary cardiovascular outcomes for women 21-year after birth (n = 3692)

	Number	Percentage %
Perceptions of health during pregnancy		
Feeling unwell (44 missing)	459	12.4
Not straight forward pregnancy (51 missing)	306	8.4
Have minor complications (61 missing)	1039	28.6
Primary cardiovascular outcomes for women 21-year after birth		
Heart disease (missing = 35)	106	2.9
Hypertension (missing = 29)	987	26.9
Stroke (missing = 30)	57	1.6

Table 3 shows that after adjusting for the mediating factors, women who did not have a straightforward pregnancy had twice the odds of being diagnosed with later heart disease compared with women who experienced a straight forward pregnancy [adjusted OR 2.0, 95 % CI 1.1–3.8; with an exposure event rate (EER) of 5.2 % (16/305) versus 2.6 % (76/2930)]. Ever having depression or anxiety, and menopausal status were the only covariates to influence the association between heart disease and not having a straight forward pregnancy. Furthermore, women who had complications, other than serious complications of pregnancy, such as pre-eclampsia and gestational diabetes, during their pregnancy were 30 % more likely to have developed hypertension 21 years later [adjusted OR 1.3, 95 % CI 1.0–1.6; EER = 25.7 % (265/1031) versus 20 % (439/2198)] (see Table 4). In the final model only two covariates remained significant: parity, and being overweight or obese in mid-life.

**Table 3:** Associations as odd ratios (95 % confidence intervals) for those women ever diagnosed with heart disease

Unadjusted event rate (ER)	Ever diagnosed with heart disease		
	Perception of health during pregnancy^a^		
	Feeling unwell N = 3239	Not straight forward pregnancy N = 3235	Minor complications N = 3224
	% (n)	% (n)	% (n)
Exposed (EER)	4.5 (20/448)	5.2 (16/305)	3.6 (37/1025)
Reference group (P_0_)	2.6 (732/2791)	2.6 (76/2930)	2.5 (55/2199)
Model	OR (95 % CI)	OR (95 % CI)	OR (95 % CI)
Unadjusted^b^	1.8 (1.1–2.9)	2.1 (1.2–3.6)	1.5 (1.0–2.2)
Adjusted for factors during early pregnancy^c^	1.5 (0.9–2.6)	2.0 (1.1–3.4)	1.4 (0.9–2.2)
Adjusted for demographics factors measured at the 21-year phase^d^	1.7 (1.0–2.9)	1.9 (1.1–3.3)	1.4 (0.9–2.2)
Adjusted for clinical measures (weight, mental health)^e^	1.7 (1.0–3.1)	2.2 (1.2–4.1)	1.4 (0.8–2.3)
Adjusted for mediating factors^f^	1.6 (0.9–2.9)	2.0 (1.1–3.8)	1.3 (0.8–2.1)

^a^Cases diagnosed with Gestational diabetes, Pre-eclampsia or hypertension during the pregnancy were excluded from the analysis (380 cases removed from *Feeling unwell*, 379 cases removed from *Not straight forward*)

^b^Reference group—women that reported no morning sickness; were not unwell during the pregnancy; had a straight-forward pregnancy; or, experienced a complication free pregnancy

^c^Adjusted for factors from early pregnancy: age, highest education, parity reported at the 1st antenatal visit

^d^Adjusted for demographic factors reported at the 21-year FU: marital status, satisfaction with marital relationship, family income

^e^Adjusted for clinical measures: BMI measured at 21-year FU, anxiety or depressive symptoms (DSSI) ever during the FU phases

^f^Adjusted for mediating factors: ever smoked cigarettes, alcohol consumption and menopausal transition reported at 21-year FU

**Table 4:** Associations as odd ratios (95 % confidence intervals) for those women ever diagnosed with hypertension

Unadjusted event rate (ER)	Ever diagnosed hypertension OR (95 % CI)		
	Perception of health during pregnancy^a^		
	Feeling unwell N = 3242	Not straight forward pregnancy N = 3238	Minor complications N = 3229
	% (n)	% (n)	% (n)
Exposed (EER)	27.7 (125/451)	27.2 (83/305)	25.7 (265/1031)
Reference group (P_0_)	20.6 (576/2791)	21.3 (625/2933)	439/2198)
Unadjusted^b^	1.5 (1.2–1.9)	1.4 (1.1–1.8)	1.4 (1.2–1.7)
Adjusted for factors during early pregnancy^c^	1.4 (1.1–1.7)	1.3 (1.0–1.7)	1.3 (1.1–1.6)
Adjusted for demographics factors measured at the 21-year phase^d^	1.4 (1.1–1.7)	1.3 (1.0–1.7)	1.2 (1.0–1.5)
Adjusted for clinical measures (weight, mental health)^e^	1.3 (0.9–2.0)	1.1 (0.6–2.0)	1.3 (1.0–1.6)
Adjusted for mediating factors^f^	1.1 (0.8–1.5)	1.1 (0.7–1.6)	1.3 (1.0–1.6)

^a^Cases diagnosed with Gestational diabetes, Pre-eclampsia or hypertension during the pregnancy were excluded from the analysis (380 cases removed from *Feeling unwell*, 379 cases removed from *Not straight forward*)

^b^Reference group—women that reported no morning sickness; were not unwell during the pregnancy; had a straight-forward pregnancy; or, experienced a complication free pregnancy

^c^Adjusted for factors from early pregnancy: age, highest education, parity reported at the 1st antenatal visit

^d^Adjusted for demographic factors reported at the 21-year FU: marital status, satisfaction with marital relationship, family income

^e^Adjusted for clinical measures: BMI measured at 21-year FU, anxiety or depressive symptoms (DSSI) ever during the FU phases

^f^Adjusted for mediating factors: ever smoked cigarettes, alcohol consumption and menopausal transition reported at 21-year FU

## Discussion

Using data from a large, prospective cohort of women, this study aimed to assess women’s self-reported perceptions of their pregnancy and their risk of cardiovascular disease 21 years on. In this study, only a minority of women were diagnosed with heart disease or stroke by the time of the 21 year follow-up, which reflects the average age of those women. The prevalence of cardiovascular disease increases markedly with age (Australian Institute of Health and Welfare (AIHW) 2012) and for women; the ‘sex protective’ effects (Pilote et al. 2007) are attenuated with both age and postmenopausal status (Dasgupta et al. 2007). Pre-menopausal women appear to be protected to some extent against cardiovascular diseases, but catch up to men between the ages 60–69 years. However, the proportion of women diagnosed as having hypertension, an independent risk factor for future heart disease, is consistent with other studies and reflects that of the general Australian female population (Barr et al. 2005).

The most important and interesting findings for this study are the robust associations found between women’s perceptions of their pregnancy (not having a straightforward pregnancy, and experiencing complications) and their health (feeling unwell) during pregnancy, with cardiovascular disease and hypertension 21 years later. After adjusting for all the mediating factors, women who perceived themselves as not having a straight forward pregnancy had twice the odds (AOR 2.0, 95 % CI 1.1–3.8) of being diagnosed with heart disease compared to women with a straight forward pregnancy. This association remained after excluding the serious pregnancy complications such as essential hypertension, pre-eclampsia and other gestational disease which are all known to be important risk factors for future cardiovascular disease (Kaaja and Greer 2005). The association remained robust, although slightly attenuated after adjusting for mental and clinical measures and potential mediating factors.

Similarly, women who perceived themselves as having complications (other than the serious complications) in their pregnancy had a 30 % increased odds (AOR 1.3, 95 % CI 1.0–1.6) of having hypertension 21 years later, after adjusting for early life factors, demographic characteristics, clinical factors and potential mediators. Including a number of factors that have previously been associated with CVD such as parity and mental health problems. A number of studies have found an association between parity and risk of CVD in women (Jacobs et al. 2012; Hardy et al. 2007; Lawlor et al. 2003). Similar association was found between number of children and CVD risk in men, although men had an overall weaker magnitude of risk than women, this association may be also be due to socioeconomic, lifestyle and genetic characteristics shared by both parents.

There may be several potential explanations for our findings. Khatun et al. (2009) suggested that women who experience symptoms during pregnancy or reported difficulties during pregnancy have real medical problems, or may also have psychosocial disorders which manifest after the birth. These difficulties may increase psychological stress and their risk for future cardiovascular disease (Weidner and Spaderna 2013). This would be consistent with the theory that “pregnancy unmasks chronic illness”, this would suggest that a women’s potential for disease can be unmasked by pregnancy thus providing a window to her long term health outlook and presenting opportunities for possible primary prevention (Kaaja and Greer 2005).

Our findings are also consistent with other studies, which have found that self-rated health is a significant predictor of objective health outcomes, including cardiovascular disease (Rutledge et al. 2010) although the magnitude of risk found here is modest. The proportion of women reporting feeling unwell during pregnancy is consistent with other population based studies (Rutledge et al. 2010) suggesting the importance of self-rated health, particularly at times when the body may be under stress such as pregnancy. This result is not surprising as the study sought to extend previous research by looking at associations that span more than 20 years. Previous research has followed up participants for up to 10 years (Rutledge et al. 2010), although this research has commonly been undertaken in clinical samples (Rutledge et al. 2010; Bosworth et al. 1999; Miilunpalo et al. 1997). To our knowledge, this is the first large scale population based cohort of women to have identified this association 21 years post pregnancy (Salomon et al. 2009). It may be as Jylha (2009) suggests that self-rated health can be best considered to represent a summary of information or a judgement about bodily conditions that are involved in biological mechanisms that lead to poorer health outcomes. Jylha (2009) postulates that self-perceptions of health represents a statistical rather than a causative predictor of mortality in that it reflects the state of the individual at a given point in time. If, as Khatun et al. suggest, pregnancy places a burden on a woman’s body that unmasks inherent weakness, these self-perceptions reflect the individual’s assessment of this.

### Limitations of study

Firstly, the current study used self-reported measures to assess both the cardiovascular outcomes 21 years post birth and self-reported perceptions of pregnancy which were asked 3–5 days post-partum. Although the measures were not validated, there is considerable evidence for the robustness of self-rated perception of general health during pregnancy (Bosworth et al. 1999; Petrie and Weinman 1997) and self-rated health is widely accepted as a valid indicator in middle-aged populations (Idler and Benyamini 1997). It seems unlikely that self-report, however, is a major bias as previous research has found relatively good agreement between self-report and interviews regarding chronic, somatic diseases (Bergmann et al. 2004). Although it is not clear how generalisable the findings are, as some aspects of health perception may vary across different populations (Bowling 2005). Secondly, the loss to follow up in the cohort over time was approximately 30 %. Non-participation at different follow-ups was likely to be associated with lower socio-economic status, and younger age of the mother at the birth of the child (i.e. <20 years of age) (Ware et al. 2006).

## Conclusion

Self-perceptions of health represent a useful form of risk assessment that can add predictive value to more traditionally assessed risk factors. In clinical practice, it may be a useful screening tool to assess an individual’s health status.

## Additional file

10.1186/s40064-015-1639-6 Bivariate associations between factors and ever diagnosed with hypertension or heart disease, n = 3692.
