# Supplementary material for: The association between perception of health during pregnancy and the risk of cardiovascular disease: a prospective study
Source: Springerplus. 2016 Jan 4;5:6. doi: 10.1186/s40064-015-1639-6 (PMC4700040; doi:10.1186/s40064-015-1639-6)
Supplement: Supplementary file 1 — 10.1186/s40064-015-1639-6 Bivariate associations between factors and ever diagnosed with hypertension or heart disease, n = 3692. [file 40064_2015_1639_MOESM1_ESM.docx]

**Additional file 1: Table S1. Bivariate associations between factors and ever diagnosed with hypertension or heart disease, n=3692**

|  | | *Women ever diagnosed with OR (95% CI)* | |
| --- | --- | --- | --- |
| *Model 1 Early pregnancy factors* | | Hypertension | Heart Disease |
| Age at study entry (years)^1^ | |  |  |
|  | 20 to 34 years | 1.1 (0.9-1.3) | 2.4 (1.1-5.6) |
|  | 35 years or more | 1.5 (1.0-2.1) | 5.1 (1.8-14.2) |
| Parity^2^ | |  |  |
|  | 1 previous pregnancy | 1.1 (0.9-1.3) | 1.4 (0.9-2.3) |
|  | 2 previous pregnancies | 1.3 (1.0-1.6) | 1.4 (0.8-2.5) |
|  | 3 previous pregnancies | 1.4 (1.1-1.8) | 2.2 (1.1-4.5) |
|  | 4 or more previous pregnancies | 1.6 (1.2-2.2) | 3.6 (1.7-7.7) |
| Maternal education ^3^ | |  |  |
|  | Incomplete high school | 1.5 (1.2-1.9) | 1.7 (0.9-3.2) |
|  | Complete high school | 1.2 (1.0-1.4) | 1.1 (0.6-1.9) |
| *Model 2 demographic factors reported at the 21-year phase* | | |  |
| Marital status^4^ | |  |  |
|  | Live in relationship | 1.1 (0.9-1.3) | 0.7 (0.4-1.0) |
| Family income ^5^ | |  |  |
|  | Low income (<$399/week) | 1.2 (1.0-1.4) | 2.4 (1.6-3.5) |
| Dyadic adjustment ^6^ | |  |  |
|  | Conflict | 0.6 (0.4-1.0) | 1.5 (0.6-3.8) |
| *Model 3 Clinical measures measured at the 21-year phase* | | |  |
| Anxiety at previous phases^7^ | |  |  |
|  | One or more episodes | 1.4 (1.2-1.6) | 2.8 (1.7-4.5) |
| Depression at previous phases ^8^ | |  |  |
|  | One or more episodes | 1.0 (0.8-1.2) | 1.1 (0.6-2.0) |
| BMI (kg/m^2^) ^9^ | |  |  |
|  | Overweight (25.0-29.9) | 2.1 (1.6-2.7) | 0.9 (0.4-1.8) |
|  | Obese (> 30.0) | 4.1 (3.2-5.3) | 1.2 (0.6-2.3) |
| *Model 4 Mediating factors measured at 21-year phase* | | |  |
| Ever smoked cigarettes ^10^ | | 0.9 (0.8-1.0) | 1.1 (0.7-1.6) |
| Alcohol consumption ^11^ | |  |  |
|  | Light drinker | 1.0 (0.8-1.2) | 0.7 (0.4-1.1) |
|  | Moderate to heavy drinker | 0.7 (0.5-0.9) | 0.6 (0.3-1.1) |
| Menopausal status at 21-year phase ^12^ | |  |  |
|  | Perimenopausal | 1.3 (1.1-1.6) | 2.3 (1.3-4.1) |
|  | Postmenopausal | 1.5 (1.2-1.9) | 2.0 (1.0-3.9) |
|  | Surgical | 1.4 (1.2-1.8) | 3.9 (2.2-7.1) |

Reference group for never diagnosed with hypertension or never diagnosed with heart disease:

1. Mothers aged 19 years or less 2. 1^st^ pregnancy 3. Post high school qualifications
2. Currently single 5. Family income of AU$400 per week or more
3. No martial conflict 7. No anxiety (as measured by DSSI) at any phase
4. No depression (as measured by DSSI) at any phase 9. Normal or underweight
5. Never smoked cigarettes 11. Abstainer 12. Perimenopausal
